# DFMA-DETR: a pomegranate maturity detection algorithm based on dual-domain feature modulation and enhanced attention

**DOI:** 10.3389/fpls.2025.1680299

**Published:** 2025-10-22

**Authors:** Xinyue Huang, Feng Song, Tanglong Feng, Yao Zhou, Wen Peng

**Affiliations:** ^1^ School of Software Engineering, Jiangxi University of Science and Technology, Nanchang, China; ^2^ International College, Nanchang Hangkong University, Nanchang, China

**Keywords:** pomegranate maturity detection, RT-DETR, attention mechanism, object detection, deep learning

## Abstract

Accurate detection of pomegranate maturity plays a crucial role in optimizing harvesting decisions and enhancing economic benefits. Conventional approaches encounter significant challenges in complex agricultural scenarios, including limited feature representation capabilities, singular attention mechanisms, and insufficient multi-scale information fusion. This study presents the DFMA-DETR algorithm, which establishes an end-to-end detection framework through dual-domain feature modulation and enhanced attention mechanisms. The core contributions include: (1) Development of the DFMB-Net backbone network that employs spatial-frequency collaborative processing to model pomegranate surface textures, color variations, and morphological characteristics. (2) Construction of the EAFF enhanced attention feature fusion module that integrates adaptive sparse attention mechanisms with multi-scale feature adapters, effectively addressing feature representation challenges under complex background interference; (3) Introduction of the AIUP adaptive interpolation upsampling processor and MFCM multi-branch feature convolution module, substantially improving feature alignment accuracy and multi-scale representation performance. Experimental validation on the constructed PGSD-5K dataset demonstrates that DFMA-DETR achieves detection accuracies of 90.23% mAP@50 and 76.40% mAP@50-95, representing improvements of 3.13% and 3.06% respectively over the baseline RT-DETR model, while maintaining relatively low model complexity. Cross-dataset validation further confirms the superior generalization performance of the proposed approach. This research provides an effective solution for advancing intelligent detection technologies in precision agriculture.

## 1 Introduction

Pomegranate, one of the healthiest and most widely eaten fruits globally, has significant repercussions for agricultural production, post-harvest management, and food production through the precise detection of maturity (Magwaza and Opara, 2015; Okere et al., 2022). The precise determination of maturity not only has direct repercussions on nutrition quality, taste characteristics, and storage life of the fruit but also serves as a significant determinant for determining optimal harvesting time, reducing post-harvest losses, and increasing economic yield. As consumer demand for high-quality pomegranates continues to grow and agricultural intelligence develops at a faster speed, there is a need to establish effective and accurate pomegranate maturity detection systems as an imperative for the innovative development of modern agricultural technology (Zhang et al., 2025a).

Traditional pomegranate maturity detection methods predominantly rely on destructive physicochemical analysis such as colorimetry, near-infrared spectroscopy, and sensory evaluation. Nicolaï et al (Nicolai et al., 2007)presented a comprehensive discussion of the application of near-infrared spectroscopy in non-destructive measurement of fruit quality in their review, demonstrating that the technology accurately measures soluble solid content but is limited by spectral penetration depth restraints and fruit peel interference issues. Traditional colorimeter methods, although capable of providing objective color measurement data, are only reflections of fruit appearance characteristics and do not thoroughly examine inherent quality differences (Beghi et al., 2017; Zhang et al., 2021). Although these methods are somewhat effective for pomegranate maturity assessment, they are operationally complex, inefficient, and unsuitable for large-scale automatic applications.

The recent years have witnessed significant progress in deep learning technologies applied in agricultural sectors, opening up new solutions for detecting pomegranate maturity. Aherwadi et al. (2022) employed convolutional neural network (CNN) technology to classify banana maturity with 81.96% accuracy and demonstrated the feasibility of fruit maturity detection using deep learning. CNN-based methods of detecting fruits have exhibited excellent performance in determining maturity in fruits like apples and mangoes (Lu et al., 2022; Zhang et al., 2025b). But these deep learning approaches based on classification primarily solve for discrimination of single fruit maturity, and the real-time detection capability in dense agricultural environments must be enhanced.

Over the past few years, computer vision domains have witnessed remarkable advancements in object detection methodologies, whose accuracy and real-time performance strengths have made them some of the leading contenders for applications in agricultural intelligence. Out of fruit maturity detection researches employing object detection architectures, algorithms from the YOLO series have been widely utilized due to their best-in-class speed-accuracy trade-off. Tian et al (Tian et al., 2019)constructed an improved YOLOv3-based apple detection algorithm for processing low-resolution feature layers in networks with significantly improved detection performance under heavy orchard scenes. Cao and Yuan (2022) constructed an improved YOLOv4 algorithm for real-time mango detection with CBAM attention mechanisms to improve small target detection efficiency and detection speed. As far as pomegranate detection is concerned, Yu et al. (2022) constructed a pomegranate fruit localization and detection system with RGB-D feature fusion in a Mask R-CNN framework, even though the approach has extremely high computational cost and is difficult to satisfy real-time detection requirements. Apart from this, SSD algorithms have also been employed for the detection of fruits, with Vasconez et al. (2020) showing that SSD is capable of higher detection accuracy at the expense of faster detection rates. Despite these object detection methods having good performance detecting fruit, they tend to be non-maximum suppression (NMS) based, which affects detection speed and accuracy.

Given the maturity and strong real-time performance of Transformer architecture-based methods in recent years, we adopt Transformer-based object detection approaches. With the successful application of Transformer architectures in computer vision domains, attention mechanism-based object detection methods have demonstrated powerful feature representation capabilities and global modeling advantages, providing novel technical pathways for real-time object detection. DETR (Detection Transformer), as the first end-to-end Transformer object detector, simplified detection processes by eliminating manually designed components. Carion et al. (2020) demonstrated the effectiveness of Transformer architectures in object detection tasks within the original DETR paper, achieving performance comparable to Faster R-CNN on the COCO dataset.

However, DETR’s high computational cost limits its real-time applications. To address this limitation, Zhao et al. (2024) proposed RT-DETR (Real-Time Detection Transformer), achieving 53.1% AP and 108 FPS with RT-DETR-R50 on the COCO dataset through efficient hybrid encoder design and IoU-aware query selection mechanisms, surpassing equivalent-scale YOLO detectors. Recent years have witnessed rapid emergence of RT-DETR-based agricultural application research. Wang S. et al. (2024) proposed a lightweight tomato maturity detection algorithm PDSI-RTDETR, reducing parameters and computational costs by 30.8% and 17.6% respectively while maintaining high accuracy. Zhang Y. et al. (2025) constructed a WMC-RTDETR lightweight tea disease detection model, effectively improving detection robustness under complex environments. Nevertheless, RT-DETR application research specifically targeting pomegranate maturity detection remains relatively limited.

Although object detection methodologies have achieved relative maturity in fruit detection domains, specific applications in pomegranate maturity detection still face numerous difficulties and challenges. Firstly, traditional convolutional neural network backbone structures can only capture local spatial features, struggling to establish long-range dependencies while lacking effective utilization of frequency domain information, making them unable to adequately model complex textural and periodic characteristic variations on pomegranate surfaces. Secondly, existing attention mechanisms demonstrate insufficient precision in weight allocation under complex agricultural environments, with singular feature fusion strategies and lack of multi-scale spatial context awareness capabilities, resulting in difficulties accurately capturing key visual features of pomegranate maturity. Furthermore, nearest neighbor interpolation upsampling methods in traditional feature pyramid networks easily generate feature misalignment and information loss, while standard convolution operations are limited to single receptive fields, making efficient multi-scale feature expression challenging.

Therefore, addressing the aforementioned issues, we propose a pomegranate maturity detection algorithm named DFMA-DETR. The contributions of this work are as follows:

1. We constructed the PGSD-5K dataset specifically designed for pomegranate maturity detection, containing 5,855 high-quality images covering five critical stages throughout plant growth processes. All images were precisely annotated using LabelImg annotation tools and converted to standard formats. The dataset encompasses plant images under different illumination conditions, shooting angles, and background environments, demonstrating excellent diversity and representativeness, providing reliable data foundations for pomegranate maturity detection model training and evaluation.
2. We designed DFMB-Net (Dual-domain Feature Modulation Backbone Network), innovatively extending traditional single spatial domain feature extraction to spatial-frequency domain collaborative processing modes. This network achieves multi-domain deep modeling of pomegranate surface textures, color variations, and morphological characteristics through organic combinations of HFCA (Hierarchical Feature Cascade Aggregator) modules, MDFP (Multi-Domain Feature Processor) modules, and MSRU and FTEU sub-modules, effectively addressing insufficient feature representation capabilities of traditional backbone networks.
3. We constructed EAFF (Enhanced Attention Feature Fusion) enhanced attention feature fusion modules through integrating DASA (Dynamic Adaptive Sparse Attention) adaptive sparse attention mechanisms, SPFN (Spatial-Parallel Feedforward Network) spatial enhancement feedforward networks, and MASR (Multi-scale Adaptive Scale Regulator) multi-scale feature adapters, achieving precise modeling of pomegranate surface textures, colors, and morphological features, effectively addressing insufficient feature expression and inaccurate attention focusing issues of traditional attention methods under complex background interference.
4. We proposed AIUP (Adaptive Interpolation Upsampling Processor) adaptive interpolation upsampling processors and MFCM multi-branch feature convolution modules. AIUP effectively addresses feature mismatch and information loss issues in traditional upsampling processes through soft neighborhood interpolation strategies and adaptive weight decay mechanisms. MFCM employs dual-branch heterogeneous architectures, significantly enhancing multi-scale feature representation capabilities while maintaining lightweight design through collaborative actions of standard convolution branches and depth enhancement branches.

## 2 Materials and methods

### 2.1 Dataset

**Figure 1:**
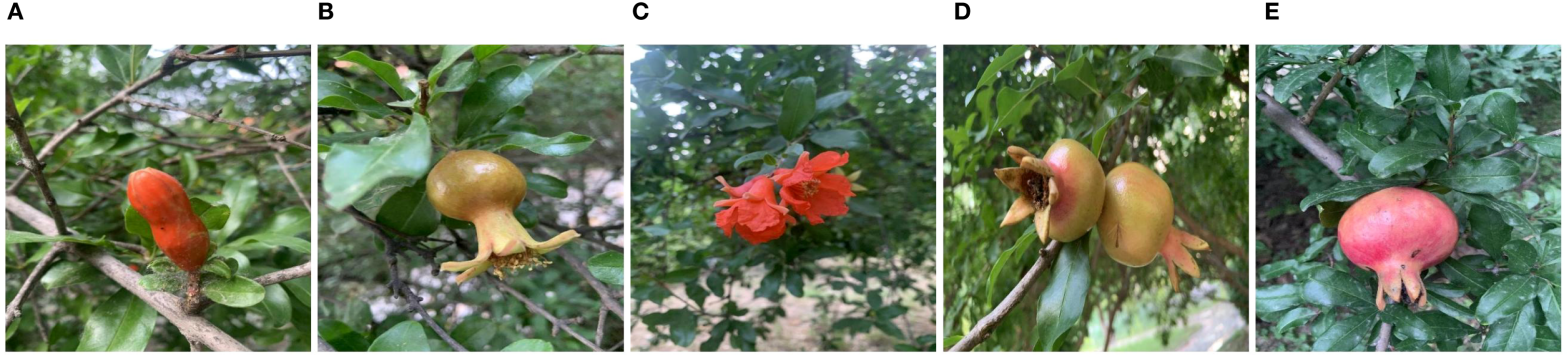
Sample images from different categories in the PGSD-5K dataset, where **(A)** Represents bud, **(B)** Represents early-fruit, **(C)** Represents flower, **(D)** Represents mid-growth, and **(E)** Represents ripe.

This study constructed the PGSD-5K dataset, specifically designed for plant growth stage object detection tasks, comprising 5,855 high-quality images. Dataset images were collected from various online data sources, encompassing five critical stages throughout plant growth processes: Bud, Early-Fruit, Flower, Mid-Growth, and Ripe, as illustrated in **Figure 1**. To ensure data quality and effective model training, all images were precisely annotated using the LabelImg annotation tool and converted to YOLOv8 standard format. During data preprocessing, automatic orientation correction was applied to each image, with all images uniformly resized to 640×640 pixel resolution using stretching methods to meet model input requirements. To guarantee training data authenticity and realism, this research employed no data augmentation techniques.

Following standard practices for deep learning model training, the dataset was partitioned into training, validation, and test sets using a 7:2:1 ratio, containing 4,098, 1,171, and 586 images respectively, ensuring independence and reliability for model training, validation, and testing procedures. Statistical analysis revealed that the entire dataset contains 8,021 annotated targets distributed across five maturity stages as follows: Bud (1,456 targets, 18.2%), Early-Fruit (1,632 targets, 20.4%), Flower (1,589 targets, 19.8%), Mid-Growth (1,678 targets, 20.9%), and Ripe (1,666 targets, 20.8%), demonstrating a balanced distribution across categories and providing sufficient sample foundations for effective model learning. The detailed class distribution statistics are presented in **Table 1**. The dataset encompasses plant images captured under diverse illumination conditions, shooting angles, and background environments, demonstrating excellent diversity and representativeness to effectively support training and evaluation of plant growth stage detection models.

**Table 1:** PGSD-5K dataset class distribution statistics.

Maturity stage	Images	Targets	Percentage	Train	Val	Test
Bud	1168	1456	18.2%	818	234	116
Early-Fruit	1306	1632	20.4%	914	261	131
Flower	1271	1589	19.8%	890	254	127
Mid-Growth	1342	1678	20.9%	939	268	135
Ripe	1331	1666	20.8%	927	265	139
Total	5855	8021	100.0%	4098	1171	586

### 2.2 RT-DETR

RT-DETR (Real-Time Detection Transformer) serves as the first real-time end-to-end object detector, employing convolutional neural networks as backbone networks for feature extraction. The backbone network typically adopts classical residual network architectures such as ResNet-18 or ResNet-50, which effectively address gradient vanishing problems in deep networks through residual connections, enabling training of deeper network structures. RT-DETR selects feature outputs from the final three stages of the backbone network as encoder inputs, with this multi-scale feature extraction strategy facilitating capture of semantic information and spatial details across different hierarchical levels.

The core innovation of RT-DETR lies in its efficient hybrid encoder design, comprising Attention-based Intra-scale Feature Interaction (AIFI) modules and CNN-based Cross-scale Feature Fusion (CCFF) modules. AIFI modules specifically process high-level features from the S5 stage of the backbone network, utilizing multi-head self-attention mechanisms to capture associations between semantic conceptual entities, thereby promoting object localization and recognition. CCFF modules integrate feature information across different scales, effectively fusing features from S3, S4, and S5 stages through cross-scale fusion strategies, enhancing the model’s detection capabilities for targets of varying sizes. In the decoder section, RT-DETR employs a transformer decoder architecture combined with auxiliary prediction heads for iterative optimization, providing high-quality initial target queries for the decoder through uncertainty minimization query selection strategies. The decoder progressively optimizes target queries through multi-layer transformer blocks, ultimately generating target category and bounding box prediction results.

### 2.3 DFMA-DETR algorithm

**Figure 2:**
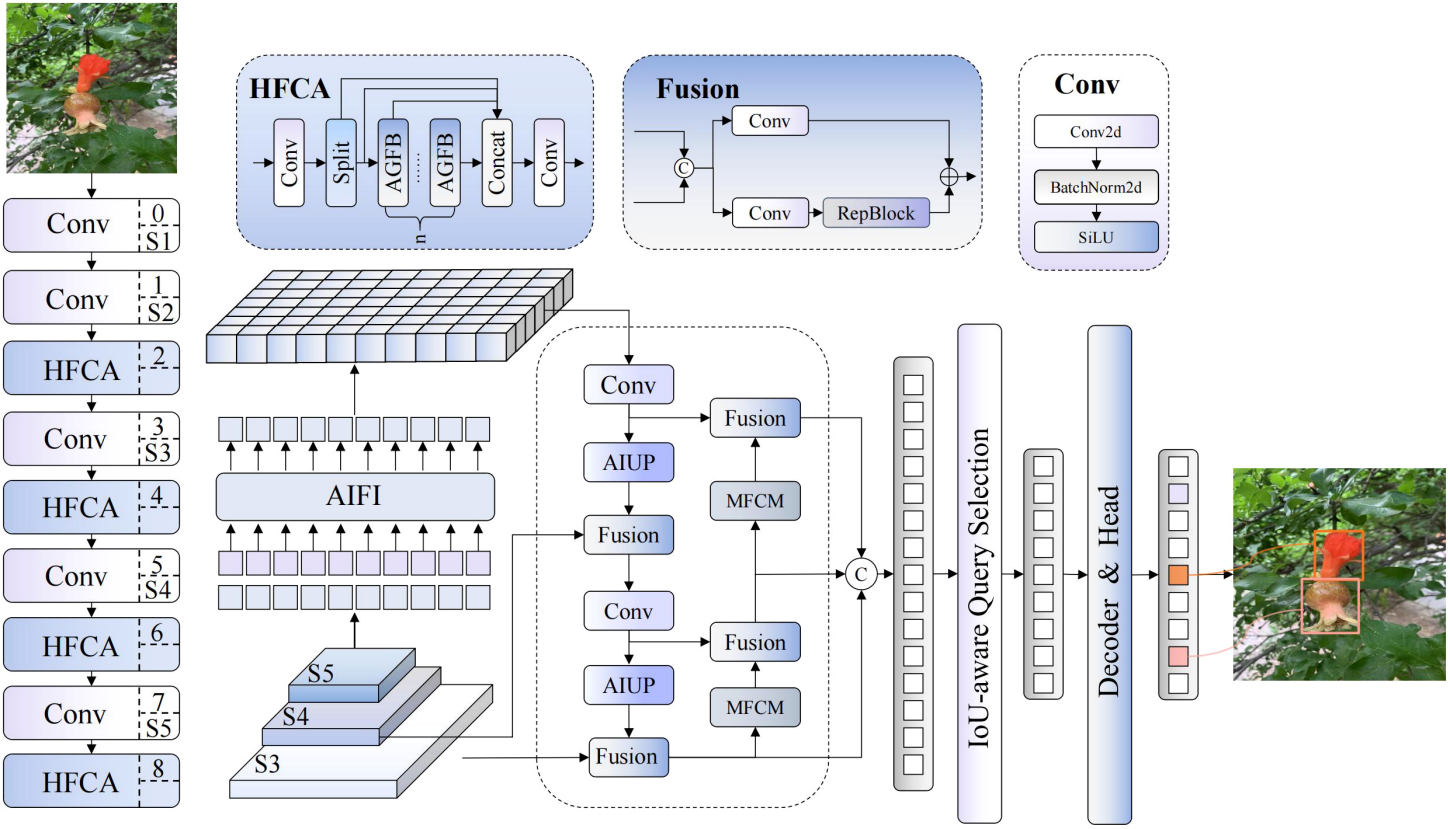
Network architecture diagram of the DFMA-DETR algorithm.

This paper proposes a pomegranate maturity detection algorithm named DFMA-DETR, whose architecture is illustrated in **Figure 2**. This algorithm addresses the limitations of traditional detection methods in complex agricultural scenarios. These limitations include insufficient feature representation capabilities, singular attention mechanisms, and limited multi-scale feature fusion effectiveness. Our solution constructs an end-to-end detection framework integrating dual-domain feature modulation and enhanced attention mechanisms. The algorithm employs DFMB-Net (Dual-domain Feature Modulation Backbone Network) as the backbone network, effectively resolving feature representation limitations of traditional networks in complex illumination conditions and multi-scale target detection through fusion of spatial-frequency dual-channel feature processing mechanisms and adaptive gated attention modules.

In the encoder section, the algorithm introduces EAFF (Enhanced Attention Feature Fusion) enhanced attention feature fusion modules, achieving precise modeling of pomegranate surface textures, colors, and morphological features through integration of adaptive sparse attention mechanisms, spatial enhancement feedforward networks, and multi-scale feature adapters. Additionally, the algorithm designs AIUP (Adaptive Interpolation Upsampling Processor) adaptive interpolation upsampling processors and MFCM multi-branch feature convolution modules, significantly improving feature alignment accuracy and multi-scale representation capabilities through soft neighborhood interpolation strategies and multi-branch collaborative processing mechanisms.

### 2.4 DFMB-Net backbone network

**Figure 3:**
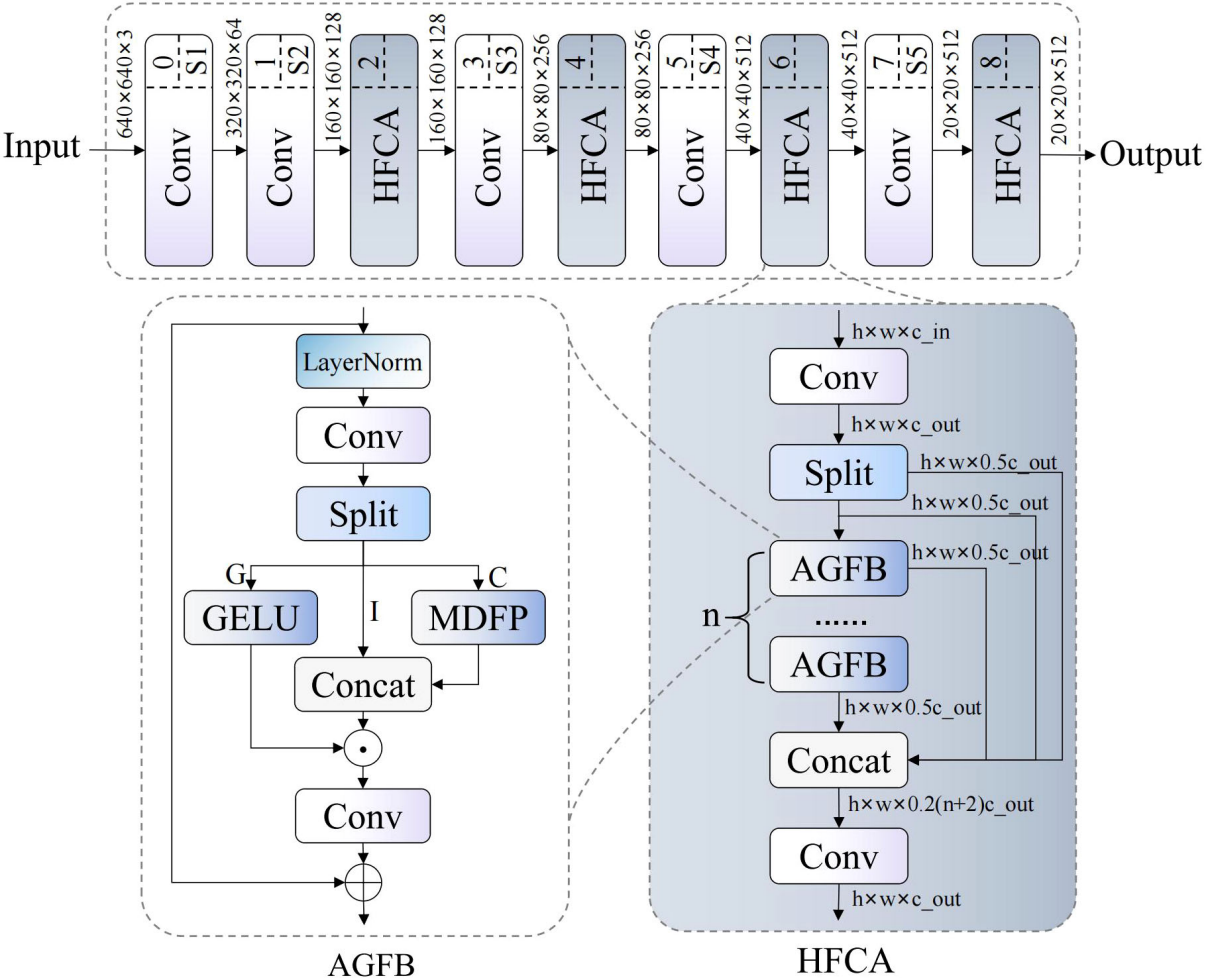
DFMB-Net network structure diagram, where ⊗ denotes element-wise addition and ⊗ denotes element-wise multiplication.

Traditional convolutional neural network backbone structures exhibit significant limitations in complex agricultural scenarios. Standard convolution operations capture only local spatial features and struggle to establish long-range dependencies. Additionally, traditional networks lack multi-scale adaptive perception, performing poorly in tasks requiring precise identification of subtle texture variations. Therefore, we propose DFMB-Net (Dual-domain Feature Modulation Backbone Network), whose structure is shown in **Figure 3**. This network addresses feature representation limitations through spatial-frequency dual-channel processing and adaptive gated attention modules, significantly improving pomegranate maturity recognition accuracy and environmental adaptability.

DFMB-Net backbone network innovatively extends traditional single spatial domain feature extraction to spatial-frequency collaborative processing modes, achieving adaptive feature selection and enhancement through HFCA (Hierarchical Feature Cascade Aggregator) modules. The network architecture employs progressive feature abstraction design, with each stage dynamically adjusting weights of different feature channels through gating mechanisms while introducing fractional-order transform theory to process frequency domain features, thereby maximizing feature representation capabilities while maintaining computational efficiency.

HFCA modules serve as core components of the DFMB-Net network, employing hierarchical feature fusion strategies to process multi-scale information. These modules first decompose input features into two parallel branches through 1×1 convolution, then utilize cascaded AGFB (Attention-Guided Feature Block) units for progressive feature extraction. The forward propagation process of the module can be expressed as:


$$\text{Y}={\text{Conv}}_{1\times 1}(\text{Concat}([{\text{Y}}_{0},{\text{Y}}_{1},\ldots ,{\text{Y}}_{n}])),{\text{ whereY}}_{i}={\text{G}}_{\text{AGFB}}({\text{Y}}_{i-1})$$

Where Y_0_ and Y_1_ represent decomposition results of input feature X through initial 1×1 convolution, ${\text{G}}_{\text{AGFB}}$ represents AGFB transformation, and n denotes the number of repetitive units within the module. This formula describes how features are progressively enhanced through recursive processing, with each Y_i_ containing information from previous layers while incorporating new feature representations, ultimately achieving effective integration of multi-level information through feature concatenation and convolution fusion.

AGFB modules implement spatial-frequency feature selection and fusion based on gating mechanisms. These modules adopt structures similar to GLU (Gated Linear Unit) (Shazeer, 2020), decomposing features into gating signals, identity mapping, and convolution processing components through grouped convolution. The core computational process is expressed as:


$$\text{G},\text{I},\text{C}=\text{Split}({\text{FC}}_{1}(\text{Norm}(\text{X})),[\text{h},\text{h}-c,c])$$

$$\text{Y}={\text{FC}}_{2}(\sigma (\text{G})⊙\text{Concat}([\text{I},{\text{F}}_{\text{MDFP}}(\text{C})]))+\text{X}$$

Where G, I, and C represent gating features, identity features, and convolution features respectively, h denotes hidden layer dimensions, c represents convolution channel numbers, σ denotes activation functions, ⊙ represents element-wise multiplication, and F_MDFP represents MDFP transformation. This gating mechanism allows networks to adaptively select important features while suppressing redundant information, ensuring effective gradient propagation through residual connections.

**Figure 4:**
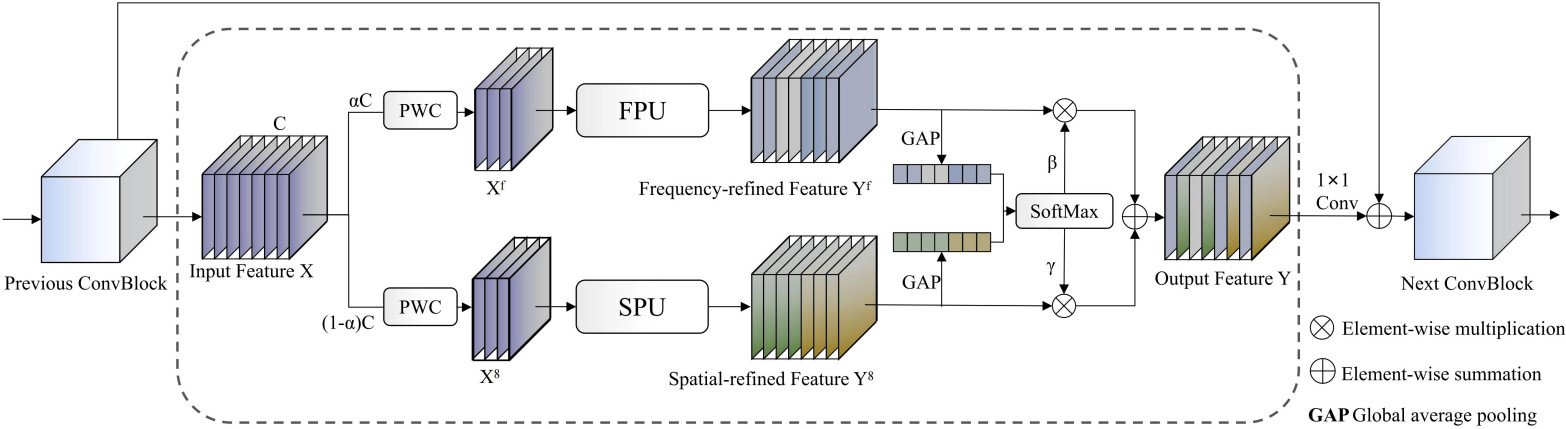
MDFP network workflow diagram.


MDFP (Multi-Domain Feature Processor) modules represent key innovative components of the network, implementing collaborative processing of spatial and frequency domain features, with workflow illustrated in **Figure 4**. These modules separately input features into MSRU (Multi-Scale Receptive Unit) and FTEU (Frequency Texture Enhancement Unit) for parallel processing, then integrate information from both domains through adaptive weight fusion mechanisms. The mathematical expression is:

$${\text{F}}_{out}={\text{PWC}}_{o}(\text{A}({\text{F}}_{spa})⊙{\text{F}}_{spa}+\text{A}({\text{F}}_{fre})⊙{\text{F}}_{fre})$$

Where ${\text{F}}_{\text{spa}}\text{=MSRU}({\text{PWC}}_{0}(\text{X}))$ and ${\text{F}}_{\text{fre}}\text{=FTEU}({\text{PWC}}_{1}(\text{X}))$ represent spatial and frequency domain processing results respectively, A(·)=Softmax(AdapAvgPool(·)) denotes adaptive attention weight computation, and PWC represents pointwise convolution operations. This design captures global periodic features through frequency domain transformation while combining local detail information from spatial domains, achieving effective fusion of multi-scale, multi-domain features particularly suitable for capturing complex texture and color variation patterns on pomegranate surfaces.

MSRU employs multi-scale receptive field adaptive adjustment mechanisms, achieving precise perception of different-scale pomegranate fruits through dynamic convolution kernels. MSRU divides input features into different channel groups, and each group adopts differently sized convolution kernels (3×3, 5×5, 7×7) for feature extraction while expanding receptive field ranges through hierarchical residual connections. The basic computation process is presented as:


$$Y_{g}^{s}=\{\begin{array}{ll} X_{g}^{s}*K_{g}, & g=1\\ (X_{g}^{s}+Y_{g-1}^{s})*K_{g}, & 1<g\leq n \end{array}$$

Where $X_{g}^{s}$ represents the g-th group input features, ${\text{K}}_{\text{g}}$ denotes corresponding convolution kernels, $Y_{g}^{s}$ represents output features, and n denotes group numbers. This recursive processing mechanism ensures each $Y_{g}^{s}$ contains information from preceding hierarchical levels while incorporating new multi-scale feature representations, ultimately achieving simultaneous capture of pomegranate fruit local texture details and global shape features through feature concatenation.

FTEU, which originates from Fractional Gabor Transform theory, is particularly adept at extracting high-frequency texture patterns and directionality of pomegranate surfaces. FTEU first applies fractional-order Fourier transforms to the input features, converting spatial domain information to frequency domain space, and then extracts texture patterns along different orientations and scales through multi-directional Gabor filter banks. The mathematical expression is:


$$K_{i,u}^{v}=K_{i,o}*G^{\alpha }(u,v)$$

Where ${\text{K}}_{\text{i,o}}$ represents learned k×k convolution kernels, $G^{\text{α}}(\text{u,v})$ denotes fractional-order Gabor filter banks with different orientations and scales, α represents transform angle parameters, and u,v represent orientation and scale indices respectively. This frequency domain analysis method effectively suppresses image noise while highlighting subtle variations in surface textures during pomegranate maturation, providing crucial frequency domain feature information for accurate maturity grade discrimination.

Through the use of the DFMB-Net backbone network, this study innovatively comes up with a multi-domain feature collaborative extraction network structure, with efficient solutions to the critical problems of deficiency of feature representation capabilities of traditional detection algorithms in complex agricultural scenes. It achieves deep merging of spatial-frequency characteristics through HFCA modules, gated attention mechanisms suitably choosing and assigning weights to salient features, as hierarchical progressive feature processing methods allow for sufficient merging and efficient utilization of multi-scale information. This new architecture not only significantly enhances network capability for pomegranate fruit maturity characteristic extraction and representation, but also significantly boosts model robustness and generalization capability under different light conditions, shooting angles, and interference scenes, bringing new theoretical foundations and technical means to intelligent detection technologies in precision agriculture.

### 2.5 EAFF module

**Figure 5:**
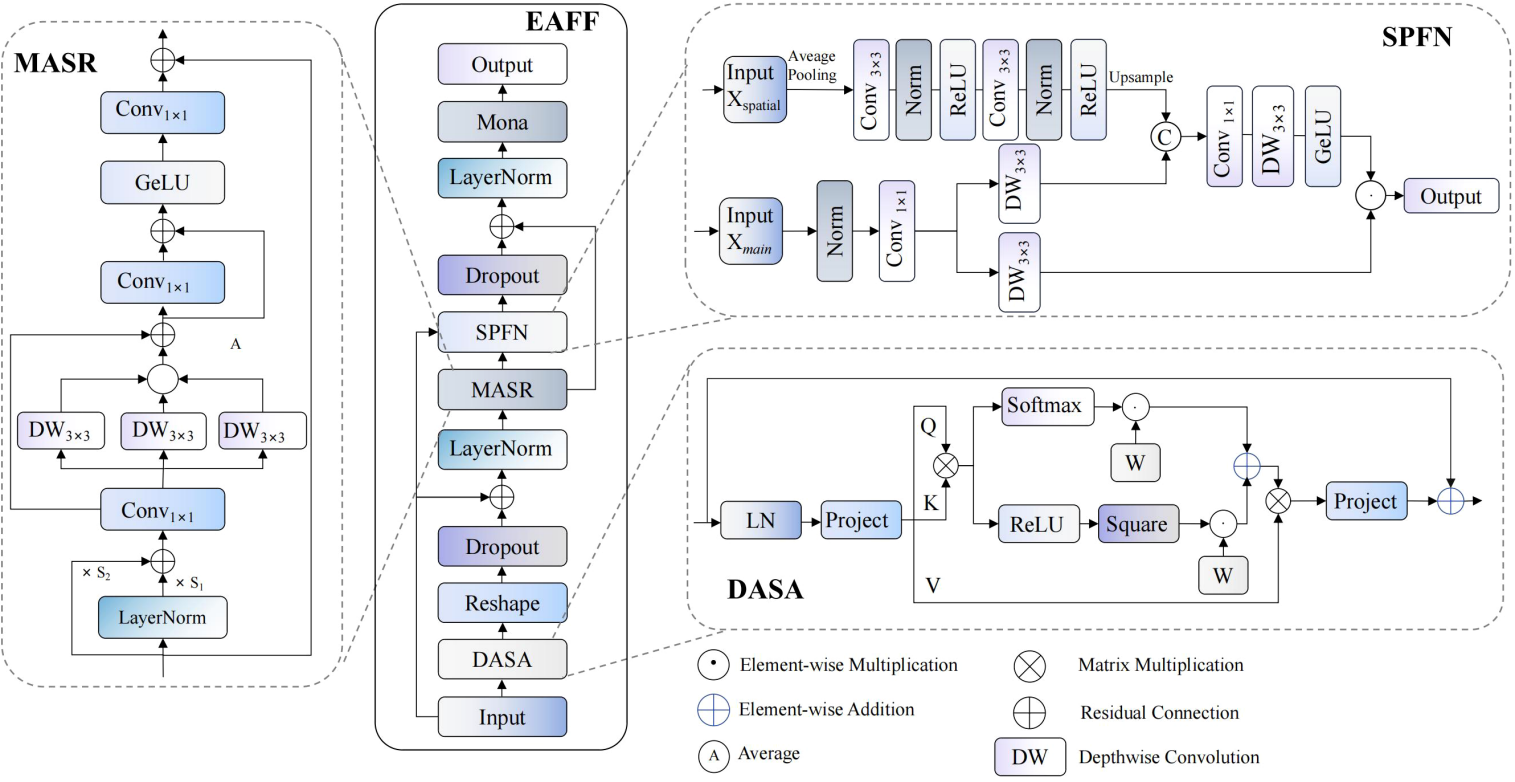
EAFF module network structure diagram.

Traditional AIFI modules exhibit significant limitations in complex agricultural scenarios: insufficient precision in attention weight allocation, singular feature fusion mechanisms, and lack of multi-scale spatial context awareness. These limitations result in difficulties capturing key visual features for pomegranate maturity detection. Therefore, we propose an enhanced encoder module named EAFF (Enhanced Attention Feature Fusion), whose structure is shown in **Figure 5**. This module achieves precise modeling of pomegranate surface textures, colors, and morphological features through integration of adaptive sparse attention mechanisms, spatial enhancement feedforward networks, and multi-scale feature adapters, effectively addressing insufficient feature expression and inaccurate attention focusing issues of traditional methods under complex background interference.

EAFF modules employ deep learning architectures combining residual connections with layer normalization, achieving progressive feature optimization through collaborative work of three core sub-modules. DASA (Dynamic Adaptive Sparse Attention) implements sparse attention computation to improve computational efficiency, SPFN (Spatial-Parallel Feedforward Network) enhances spatial context awareness through dual-path parallel processing, while MASR (Multi-scale Adaptive Scale Regulator) modules achieve adaptive feature adjustment through multi-scale convolution operations. This design innovatively combines sparse attention mechanisms with spatial enhancement techniques, constructing an efficient and precise feature expression framework.

Initially, input feature maps undergo adaptive sparse attention computation through DASA modules, with this process combining layer normalization and feature adjustment from the first MASR module, expressible as:


$${\text{Z}}_{1}={\text{MASR}}_{1}(\text{LN}(\text{X}+\text{DASA}(\text{X})))$$

Where $\text{X}\in {\text{R}}^{\text{B×C×H×W}}$ represents input feature maps, with B denoting batch size, C representing channel numbers, and H and W representing feature map height and width respectively; LN(·) denotes layer normalization operations for stabilizing training processes and accelerating convergence; DASA(·) represents adaptive sparse attention computation functions, achieving dynamic balance between sparse and dense attention through dual-branch parallel processing; MASR_1_(·) represents the first multi-scale adaptive feature adjustment module, implementing refined feature processing through parallel multi-kernel convolution and gating mechanisms.

Subsequently, preliminarily processed features enter SPFN modules for spatially enhanced feedforward processing, with this stage similarly combining layer normalization and adaptive adjustment from the second MASR module:


$${\text{Z}}_{2}=\text{MASR}(\text{LN}({\text{Z}}_{1}+SPFN))$$

Where Z_1_ represents intermediate feature representations after first-stage processing; SPFN(·,·) represents spatial enhancement feedforward network functions employing dual-path parallel processing mechanisms, with the first parameter representing current feature states and the second parameter representing original input features, achieving multi-dimensional feature optimization through collaborative action of spatial enhancement paths and main feature processing paths; MASR_2_(·) represents the second multi-scale adaptive feature adjustment module responsible for final refined adjustment of fused features; Z_2_ represents final output feature representations of the module.

DASA modules, based on core concepts of adaptive sparsity adjustment, implement dynamic balance between sparse and dense attention through dual-branch parallel processing architectures. Upon receiving windowed input features, these modules first generate query $\text{Q}\in {\text{R}}^{\text{N×d}}$ , key $\text{K}\in {\text{R}}^{\text{N×d}}$ , and value $\text{V}\in {\text{R}}^{\text{N×d}}$ matrices through linear transformations, where N=W² represents token numbers within each window and d denotes head dimensions. Sparse branches employ squared ReLU activation functions to filter attention weights with negative correlations, effectively eliminating interference from irrelevant regions; dense branches maintain traditional softmax normalization mechanisms, ensuring probabilistic properties of attention distributions. Adaptive weighted fusion processes of both branches achieve dynamic adjustment through learnable parameters, with mathematical formulation as:


$$\begin{array}{c} {\text{A}}_{ASSA}=\frac{e^{\alpha_{1}}}{e^{\alpha_{1}}+e^{\alpha_{2}}}\cdot {\text{ReLU}}^{2}(\frac{{\text{QK}}^{T}}{\sqrt{d}}+\text{B})+\frac{e^{\alpha_{2}}}{e^{\alpha_{1}}+e^{\alpha_{2}}}\\ \cdot \text{Softmax}(\frac{{\text{QK}}^{T}}{\sqrt{d}}+\text{B}) \end{array}$$

Where α_1_ and α_2_ represent learnable branch weight parameters initialized to 1 for ensuring training stability during initial phases; $\text{B}\in {\text{R}}^{\text{N×N}}$ represents relative position bias matrices achieving position awareness through two-dimensional coordinate encoding. This adaptive weight mechanism enables modules to dynamically adjust sparsity levels according to input feature complexity and task requirements, ensuring both computational efficiency improvements and maintaining integrity of key feature information.

SPFN modules follow double-path parallel processing design principles to achieve multi-dimensional optimization of input features from cooperative operation of main feature processing paths and spatial enhancement paths. Spatial enhancement paths construct global receptive field spatial context representations as a sequence of average pooling downsampling, multi-layer convolution processing, and bilinear upsampling operations of input spatial reference features. Main processing streams conduct local refinement on existing features using depthwise separable convolution and gating mechanisms. Essential computation workflows of such modules are representable as:


$${\text{X}}_{\text{SPFN}}=\text{GELU}(\text{Conv}(\text{DW}(\text{Fusion}(\text{DW}({\text{F}}_{main}),{\text{F}}_{spatial}))))⊙\text{DW}({\text{F}}_{main})$$

Where ${\text{F}}_{\text{spatial}}\text{=Upsample}({\text{Conv}}_{\text{seq}}(\text{AvgPool}({\text{X}}_{\text{spatial}})))$ represents spatial enhancement features, with ${\text{Conv}}_{\text{seq}}$ denoting two consecutive 3×3 standard convolution layers, each followed by layer normalization and ReLU activation functions. ${\text{F}}_{\text{main}}$ represents main path features processed through layer normalization and 1×1 convolution. Fusion(·,·) represents feature fusion operations, ⊙ denotes element-wise products, and DW(·) represents processing through 3×3 depthwise separable convolution operations. This design effectively combines global spatial context information with local detail features, enhancing network modeling capabilities for complex texture patterns on pomegranate surfaces.

MASR modules, based on basic rules of multi-scale adaptive feature adaptation, perform enhanced feature processing in parallel multi-kernel convolution and gating operations. Such modules first carry out adaptive normalization processing on input feature maps, adaptively scaling feature distributions using learnable scaling parameters, followed by utilizing multi-branch parallel convolution architectures to obtain spatial context information from different receptive fields. With input feature maps $\text{X}\in {\text{R}}^{\text{C×H×W}}$ , complete transformation processes of MASR modules are expressible through the following composite functions:


$${\text{F}}_{MASR}(X)=X+{\text{Φ}}_{up}(\text{GELU}(\text{Dropout}({\text{A}}_{ms}({\text{Φ}}_{down}({\text{N}}_{adapt}(X))))))$$

Where ${\text{N}}_{\text{adapt}}(\text{X})\text{=LayerNorm}(\text{X})⊙\text{γ+X}⊙{\text{γ}}_{\text{x}}$ represents adaptive normalization operations, with ${\text{γ,γ}}_{\text{x}}\in {\text{R}}^{C\times 1\times 1}$ denoting learnable scaling parameters; ${\text{Φ}}_{\text{down}}$ and ${\text{Φ}}_{\text{up}}$ represent dimensionality reduction and expansion through 1×1 convolution transformations respectively; ${\text{A}}_{\text{ms}}$ defines multi-scale adaptive aggregation operators that capture spatial features across different granularities through parallel multi-scale depthwise convolution branches while employing adaptive weight fusion strategies for effective feature integration:


$${\text{A}}_{ms}(F)=F+\frac{1}{\left|\text{K}\right|}\sum_{k\in \text{K}}{\text{DWConv}}_{k}(F;W_{k})+{\text{Φ}}_{proj}(F_{ms})$$

Where K={3,5,7} represents multi-scale convolution kernel sets, ${\text{DWConv}}_{\text{k}}$ denotes depthwise separable convolution operations with kernel sizes k×k, and ${\text{Φ}}_{\text{proj}}$ represents feature projection functions. Employment of depthwise separable convolution not only reduces computational complexity but also enhances model spatial locality modeling capabilities, particularly suitable for processing irregular shapes and complex textures in agricultural scenarios.

The proposed EAFF module constructs an efficient and precise feature expression framework through organic fusion of adaptive sparse attention mechanisms, spatial enhancement feedforward networks, and multi-scale feature adaptation techniques. Innovation of this module lies in introducing dual-branch parallel sparse attention mechanisms, achieving optimal balance between computational efficiency and feature representation capabilities through dynamic weight adjustment, while designing spatially enhanced feedforward network architectures that effectively combine global spatial context information with local detail features.

### 2.6 AIUP and MFCM modules

Traditional nearest neighbor interpolation upsampling methods in feature pyramid networks easily generate feature misalignment and information loss issues, while standard convolution downsampling operations are often limited to single receptive fields, making effective capture of multi-scale texture features and spatial context information challenging. Therefore, we propose AIUP (Adaptive Interpolation Upsampling Processor) and MFCM (Multi-branch Feature Convolution Module). AIUP modules implement soft feature alignment through introduction of adaptive scaling factors, effectively alleviating feature mismatch problems during upsampling processes; MFCM modules generate diverse receptive fields and rich texture feature representations within single modules through multi-branch convolution structures and channel shuffling mechanisms, significantly enhancing network feature representation capabilities.

Core innovations of AIUP and MFCM modules lie in transforming traditional hard interpolation and single convolution operations into adaptive soft interpolation and multi-branch collaborative processing mechanisms. AIUP modules achieve progressive feature alignment through soft neighborhood interpolation strategies, while MFCM modules significantly enhance feature diversity and expressiveness while maintaining computational efficiency through organic combination of grouped convolution, depthwise convolution, and channel shuffling, providing more precise and robust feature representations for pomegranate maturity detection.

**Figure 6:**
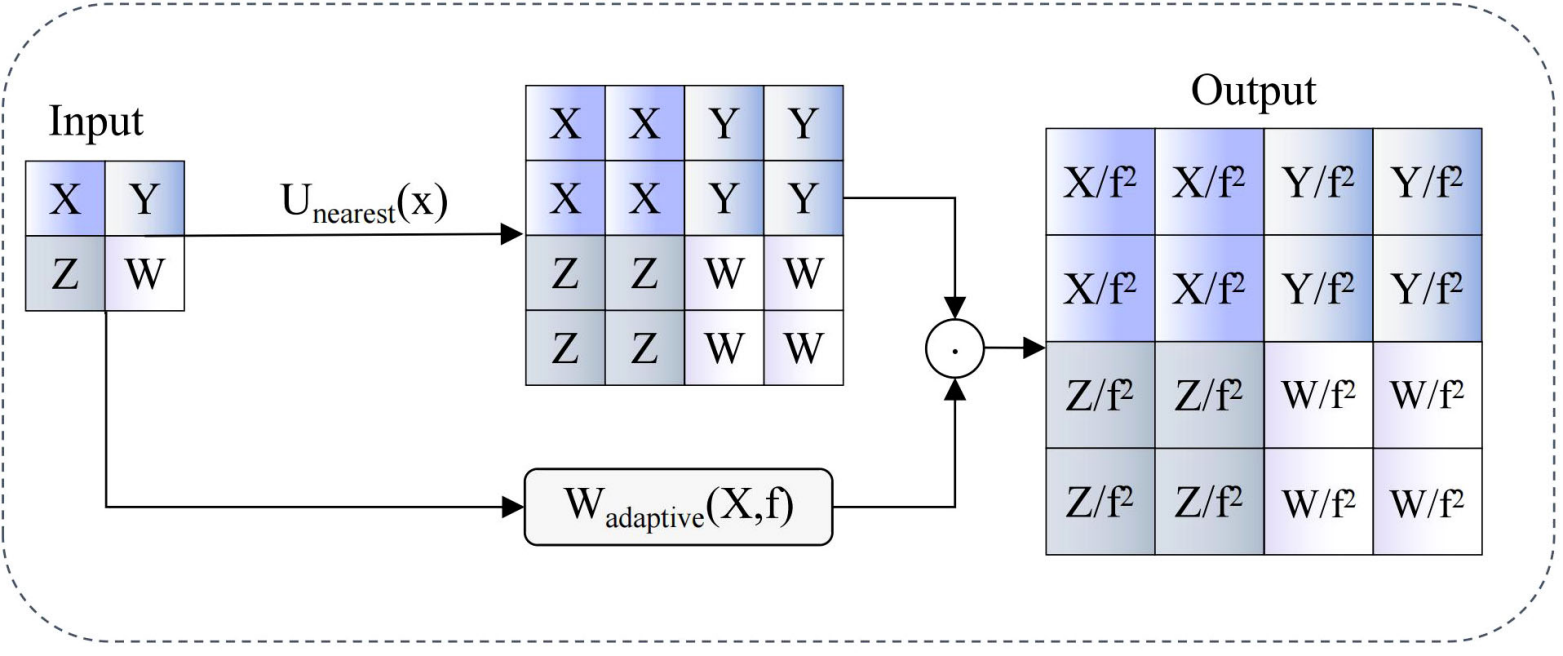
AIUP module workflow diagram.


AIUP modules employ soft neighborhood interpolation strategies, optimizing traditional upsampling processes through introduction of adaptive weight decay mechanisms, with workflows illustrated in **Figure 6**. These modules first perform nearest neighbor interpolation operations on input feature maps, then apply adaptive scaling factors related to upsampling multiples for feature modulation, thereby achieving soft feature alignment. Mathematical expressions are:

$${\text{F}}_{SNI}=\frac{1}{f^{2}}\cdot {\text{U}}_{nearest}(\text{X})⊙{\text{W}}_{adaptive}(\text{X},f)$$

Where $\text{X}\in {\text{R}}^{\text{B×C×H×W}}$ represents input feature maps, f denotes upsampling multiples, ${\text{U}}_{\text{nearest}}(\cdot )$ represents nearest neighbor interpolation operators, ${\text{W}}_{\text{adaptive}}(\cdot )$ represents adaptive weight functions, and ⊙ denotes element-wise multiplication operations. This design effectively suppresses feature distortion and noise amplification effects in traditional hard interpolation processes while maintaining computational efficiency through dynamic interpolation weight adjustment. Introduction of adaptive weight functions enables networks to automatically adjust interpolation intensities according to feature map contents, achieving smoother and more accurate feature transmission across different scales.

**Figure 7:**
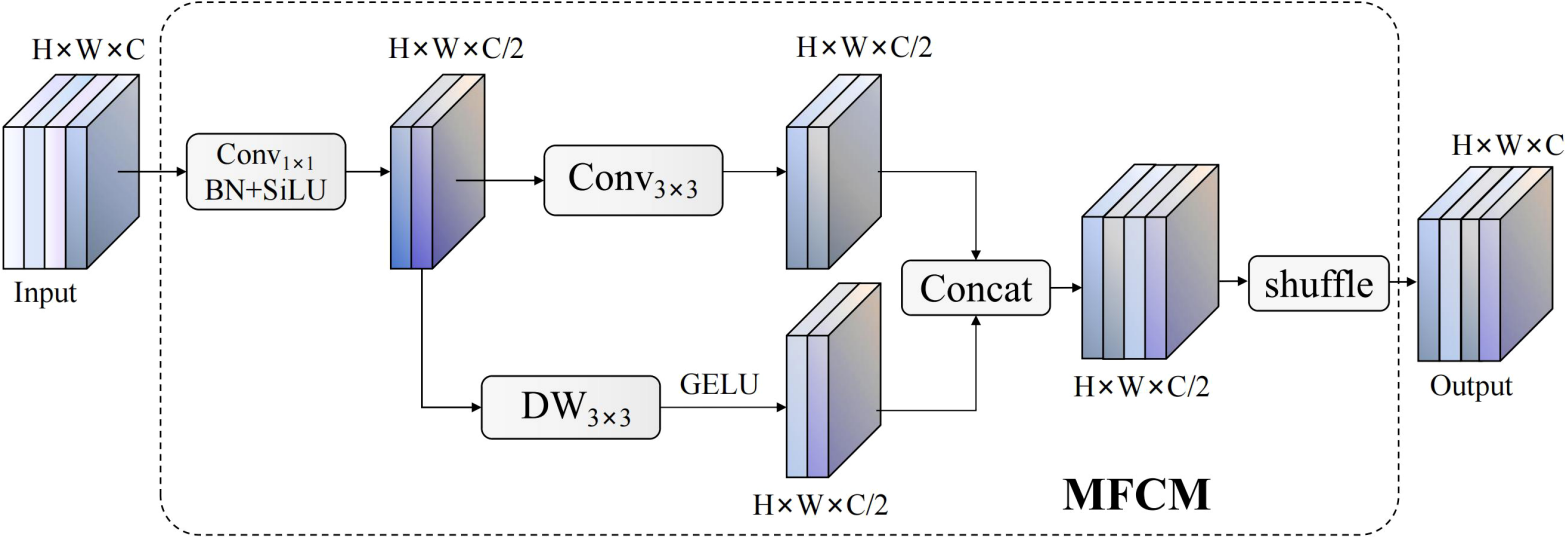
MFCM module network structure.


MFCM modules employ innovative dual-branch heterogeneous architectures, achieving efficient multi-scale feature representation learning through collaborative action of standard convolution branches and depth enhancement branches, with structures shown in **Figure 7**. Module designs fully consider balance between computational efficiency and feature richness, significantly enhancing feature extraction capabilities while maintaining lightweight characteristics through strategies of channel splitting, parallel processing, and feature reorganization. Overall transformation processes of modules are expressible through the following composite functions:

$${\text{Y}}_{GSConvE}={\text{S}}_{c\text{h}annel}([{\text{G}}_{GELU}({\text{DW}}_{3\times 3}({\text{C}}_{3\times 3}({\text{B}}_{standard}(\text{X})))),{\text{B}}_{standard}(\text{X})])$$

In this expression, ${\text{B}}_{\text{standard}}(\text{X})\text{=σ}(\text{BN}({\text{Conv}}_{\text{k×k}}(\text{X})))$ represents basic feature extraction processes of standard convolution branches, including composite operations of convolution, batch normalization, and activation functions. ${\text{C}}_{3\times 3}$ and ${\text{DW}}_{3\times 3}$ represent 3×3 standard convolution and 3×3 depthwise separable convolution operations respectively, ${\text{G}}_{\text{GELU}}$ represents GELU activation functions, [·,·] represents feature concatenation operations along channel dimensions, and ${\text{S}}_{\text{channel}}$ represents channel shuffling transformations. Channel shuffling operations achieve deep information interaction between different branch features through tensor rearrangement, with transformation matrices expressible as ${\text{P}}_{\text{shuffle}}\in {\{0,1\}}^{{\text{C}}_{\text{out}}{\text{×C}}_{\text{out}}}$ , ensuring uniform distribution and effective fusion among feature channels.

Cooperative design of AIUP and MFCM modules contributes significantly to feature alignment precision and multi-scale representation capability of RT-DETR networks. AIUP modules effectively handle feature mismatch issues in traditional upsampling processes by adaptive soft interpolation mechanisms, significantly improving feature alignment quality with different scales; MFCM modules significantly enhance the network feature capture performance and expression richness with lightweight design ensured by multi-branch cooperative processing and intelligent channel shuffling strategies. Organic combination of the two modules ensures enhanced detection networks to be more robust and accurate in confronting complex light conditions and varying pomegranate morphologies, having good technical basis for correct pomegranate maturity determination.

## 3 Experiments

### 3.1 Experimental environment and parameter settings

Experiments were conducted on a server equipped with Intel(R) Xeon(R) CPU E5–2680 v4 @ 2.40GHz 7-core processor and 64GB DDR4 memory. Deep learning training utilized NVIDIA GeForce RTX 4090 GPU (24GB VRAM) for accelerated computation. The experimental environment was based on Ubuntu 20.04.6 LTS operating system, with CUDA version 11.8 and cuDNN version 8.6.0. The deep learning framework employed PyTorch 2.0.1 in conjunction with torchvision 0.15.2 for model construction and training. Additionally, experiments utilized Python 3.9.16 as the programming language, integrating OpenCV 4.7.1 for image preprocessing, NumPy 1.24.3 for numerical computation, along with other essential scientific computing libraries.

Throughout model training, batch size was configured to 8, with input image dimensions uniformly adjusted to 640×640 pixels. The optimizer employed AdamW with initial learning rate set to 0.0001, momentum parameter configured to 0.9, and weight decay set to 0.0001. Total training epochs were established at 300 rounds. All remaining configurations followed RT-DETR default settings.

### 3.2 Evaluation metrics

This research employed the standard evaluation indexes in the object detection task to test the performance of the improved RT-DETR algorithm from various perspectives. Precision (P) refers to the proportion of correctly predicted positive samples among all predicted positive samples, and Recall (R) is the proportion of correctly predicted samples among all positive samples. mAP@0.5 (mean Average Precision at IoU=0.5) calculates the mean AP values over categories at IoU threshold 0.5, the most commonly used metric for object detection evaluation. mAP@0.5:0.95 is the mean AP values over a range of thresholds from 0.5 to 0.95 with 0.05 increments, providing more comprehensive evaluation of model detection precision. Furthermore, to determine model computational complexity and viability, GFLOPS (Giga Floating Point Operations Per Second) estimates computation load and Params (Parameters) tallies total model parameters. The metrics enable multifaceted performance evaluation of the improved algorithm regarding detection accuracy, computational efficiency, and model complexity, respectively, to guarantee practicability for real-world usage and enhance detection accuracy.

### 3.3 Ablation studies

#### 3.3.1 DFMB-Net backbone network ablation study

In order to prove the effectiveness of our proposed DFMB-Net backbone network, ablation experiments were conducted whose results are presented in **Table 2**. The experiments had rigorously tested the contribution of each element towards overall detection performance by removing HFCA modules, AGFB modules, and MDFP modules individually. All experiments were performed under identical experimental conditions and hyperparameters in order to ensure fairness and comparability of results.

**Table 2:** AVN backbone network ablation study results.

Model	HFCA	AGFB	MDFP	mAP@50	mAP@P50-95	FLOPs	Parameters
RT-DETR	×	×	×	87.10%	73.34%	57.0G	19.8M
w/o MDFP	✓	✓	×	87.45%	74.21%	48.7G	13.9M
w/o AGFB	✓	×	✓	87.21%	73.89%	49.8G	14.2M
w/o HFCA	×	✓	✓	86.98%	73.67%	51.1G	15.1M
DFMB-Net	✓	✓	✓	87.93%	74.93%	50.3G	14.7M

“✓” indicates applicable, “×” indicates not applicable.

Experimental results demonstrate that each module makes important contributions to detection performance with synergistic effects evident. Removing the MDFP module resulted in 0.48% and 0.72% decreases in mAP50 and mAP@50–95 respectively, validating the crucial role of multi-domain feature processing mechanisms in spatial-frequency collaborative modeling. AGFB module removal caused 0.72% and 1.04% decreases in mAP@50 and mAP@50–95 respectively, confirming the importance of attention-guided gating mechanisms in complex texture feature extraction. HFCA module removal yielded the most significant performance degradation, with mAP@50 and mAP@50–95 decreasing by 0.95% and 1.26% respectively, indicating that hierarchical feature cascade aggregation mechanisms constitute core components of the entire architecture.

**Figure 8:**
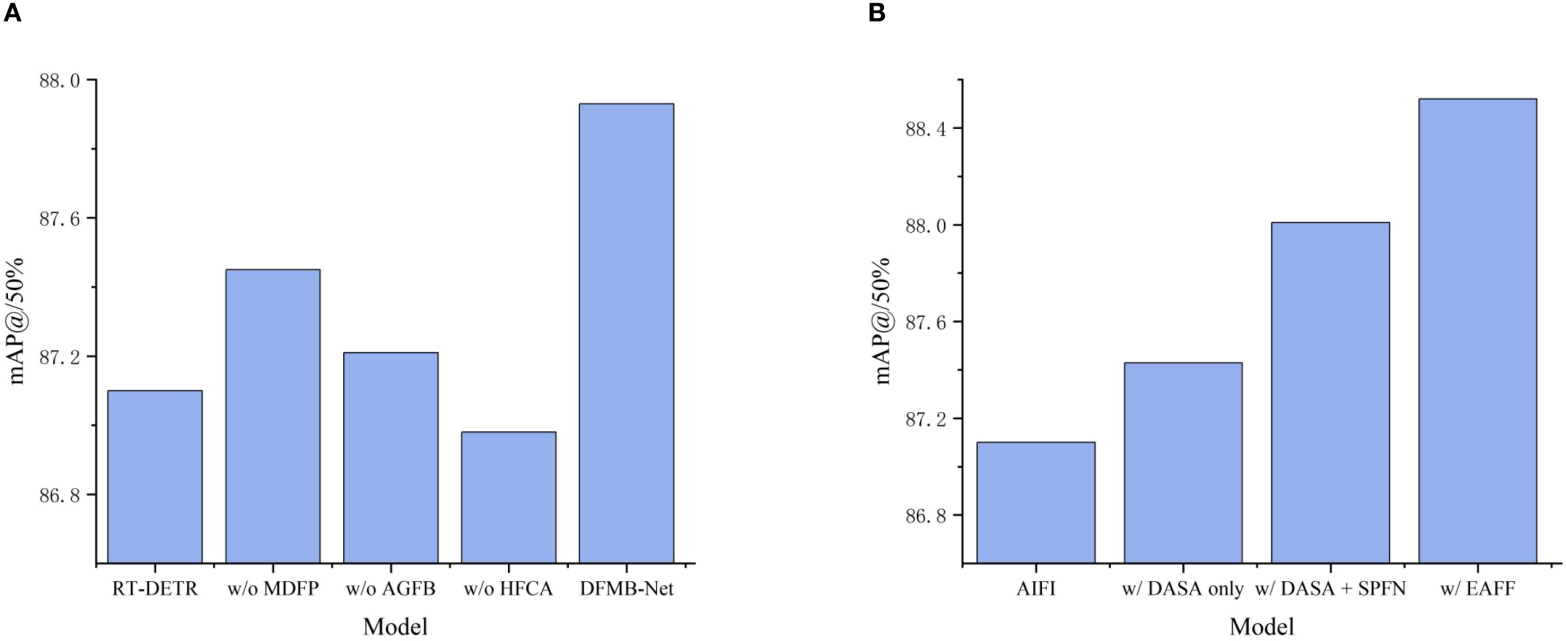
Bar charts of different experimental results, where **(A)** Shows DFMB-Net backbone network ablation study mAP@50 bar chart, and **(B)** Shows EAFF module ablation study mAP@50 bar chart.

To more intuitively demonstrate the contribution of each module to detection performance, specific performance of the DFMA-DETR algorithm under different configurations on the mAP@50 metric is illustrated in **Figure 8A**. Notably, the complete DFMB-Net backbone network not only significantly improved detection accuracy compared to the original RT-DETR baseline but also effectively reduced computational complexity and model parameters, fully validating the effectiveness of our proposed lightweight design strategy and excellent synergistic effects among modules.

#### 3.3.2 EAFF module ablation study

To validate the effectiveness of our proposed EAFF module, we conducted ablation experiments on the EAFF module, with results shown in **Table 3**. These experiments employed module replacement strategies, substituting the original AIFI modules in the RT-DETR encoder with our proposed EAFF modules. To analyze the contributions of various EAFF components in depth, we designed progressive validation experiments, sequentially verifying the independent effects of DASA adaptive sparse attention mechanisms, SPFN spatial parallel feedforward networks, and MASR multi-scale adaptive regulators.

**Table 3:** EAFF module ablation study results.

Method	DASA	SPFN	MASR	mAP@50	mAP@P50-95	FLOPs	Parameters
AIFI	×	×	×	87.10	73.34	57.0	19.8
w/DASA only	✓	×	×	87.43	73.89	57.8	20.3
w/DASA + SPFN	✓	✓	×	88.01	74.76	58.9	21.6
w/EAFF	✓	✓	✓	88.52	75.61	60.0	22.5

“✓” indicates applicable, “×” indicates not applicable.

Experimental results prove that introducing DASA mechanism alone to replace existing attention computation improved mAP50 and mAP@50–95 by 0.33% and 0.55% respectively, validating the effectiveness of adaptive sparse attention in pomegranate maturity feature capture. To more intuitively demonstrate the progressive performance enhancement effects of EAFF module components, comparison results of mAP@50 metrics under different configurations are shown in **Figure 8B**. Further integration of the SPFN module significantly improved performance, with mAP@50 and mAP@50–95 reaching 88.01% and 74.76% respectively, proving the important role of spatial parallel processing strategies in complex texture feature modeling. The complete EAFF module achieved 88.52% mAP@50 and 75.61% mAP@50–95 through multi-scale adaptive adjustment of MASR components. Compared to the original AIFI module, the EAFF module improved mAP@50 and mAP@50–95 by 1.42% and 2.27% respectively, with Recall increasing by 1.27%, fully validating that the proposed enhanced attention feature fusion module can significantly improve overall pomegranate maturity detection performance after replacing traditional AIFI.

#### 3.3.3 DFMA-DETR algorithm ablation study

To validate the effectiveness of our proposed DFMA-DETR algorithm, we designed comprehensive ablation experiments to analyze the contribution of each innovative module to detection performance, with results presented in **Table 4**. Ablation experiments were conducted on the PGSD-5K dataset, evaluating the impact on pomegranate maturity detection accuracy through progressive addition and combination of different innovative modules, where A, B, and C represent DFMB-Net dual-domain feature modulation backbone network, EAFF enhanced attention feature fusion module, and AIUP adaptive interpolation upsampling processor with MFCM multi-branch feature convolution module respectively.

**Table 4:** DFMA-DETR algorithm ablation study results.

Model	mAP@50	mAP@P50-95	Precision	Recall	FLOPs	Parameters
Base	87.10%	73.34%	91.05%	79.28%	57.0G	19.8M
A	87.93%	74.93%	90.80%	80.32%	50.3G	14.7M
B	88.52%	75.61%	91.61%	80.55%	60.0G	22.5M
C	88.21%	75.04%	91.77%	80.59%	58.1G	19.9M
A+B	89.07%	76.75%	91.28%	81.34%	51.7G	17.1M
B+C	89.35%	76.88%	92.19%	81.53%	59.4G	22.2M
A+C	88.82%	76.29%	91.41%	81.37%	52.8G	16.0M
A+B+C	90.23%	76.40%	91.63%	82.39%	51.1G	16.8M

Experimental results indicate that each innovative module significantly improves detection performance. The DFMB-Net backbone network improved mAP@50 from baseline 87.10% to 87.93% while reducing parameters from 19.8M to 14.7M, validating the effectiveness of dual-domain feature modulation mechanisms in enhancing feature representation capabilities while achieving model lightweighting. The EAFF module improved mAP@50 to 88.52%, proving the advantages of enhanced attention mechanisms in feature fusion within complex agricultural scenarios. AIUP and MFCM modules achieved 91.77% precision, demonstrating outstanding effectiveness of adaptive upsampling and multi-branch convolution in feature alignment.

**Figure 9:**
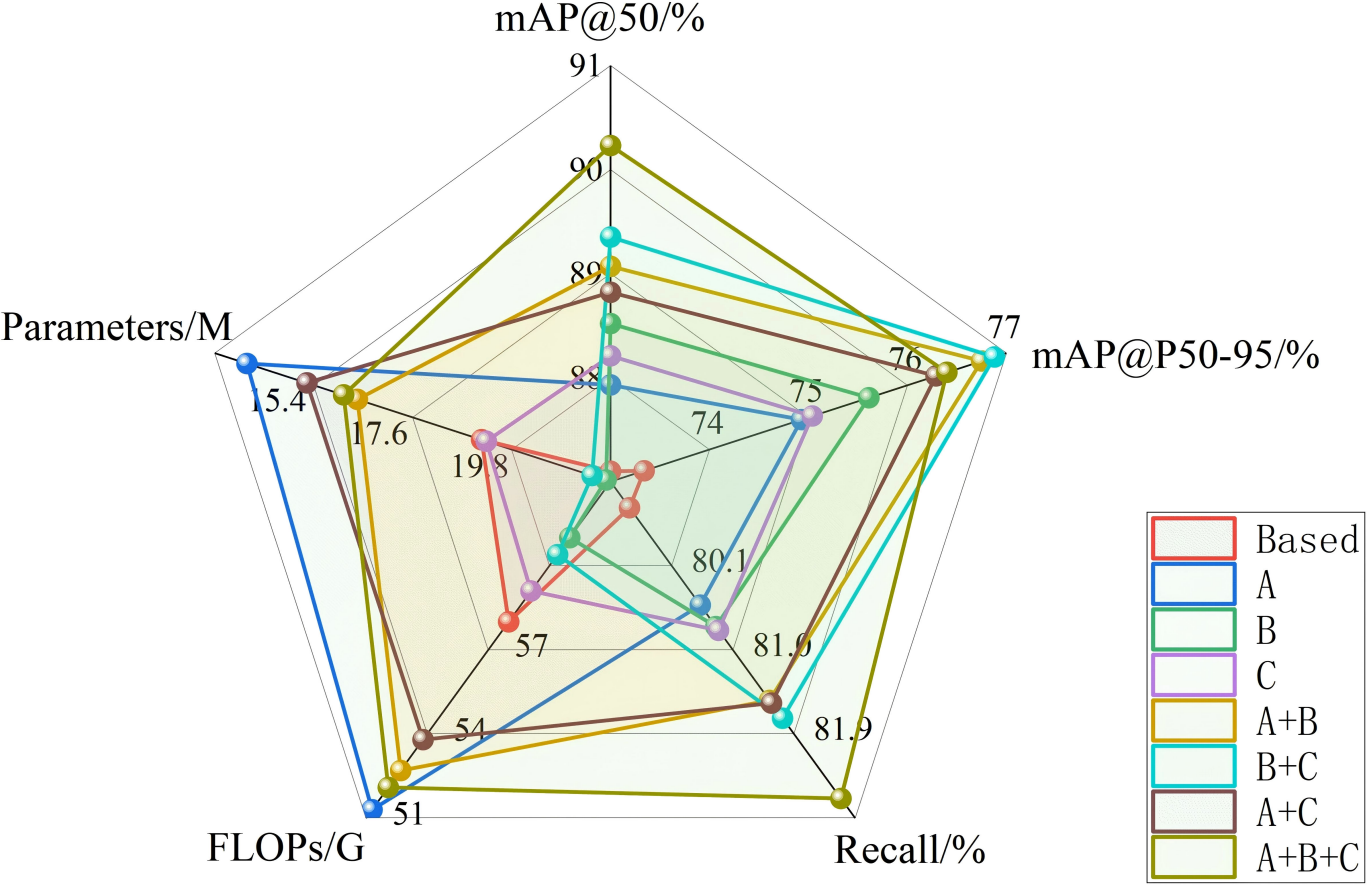
Radar chart of ablation study results.

To comprehensively evaluate the overall performance of different module combinations in detection accuracy and computational efficiency, **Figure 9** presents radar chart comparative analysis of various configurations across five key metrics: mAP@50, mAP@50-95, Recall, FLOPs, and Parameters. The radar chart intuitively reveals that the complete DFMA-DETR algorithm not only excels in detection accuracy metrics but also demonstrates excellent optimization effects in computational efficiency, with overall radar chart contours exhibiting ideal distribution characteristics of high accuracy and superior efficiency. When three modules work collaboratively, mAP@50, mAP@50-95, and recall reached 90.23%, 76.40%, and 82.39% respectively, representing improvements of 3.13%, 3.06%, and 3.11% over baseline while maintaining relatively low computational complexity and parameter count, fully validating the effectiveness and practicality of the proposed method.

### 3.4 Comparative experiments

#### 3.4.1 Stage-wise performance analysis

To provide comprehensive insights into algorithm effectiveness across different growth phases and address potential performance disparities that may be masked by averaged metrics, we conducted detailed stage-wise performance analysis on the PGSD-5K dataset. **Tables 5** and **6** present the detection performance of baseline RT-DETR and the proposed DFMA-DETR across all five pomegranate maturity stages respectively. This fine-grained evaluation reveals significant variations in detection accuracy among different growth phases, demonstrating the critical importance of stage-specific analysis in agricultural object detection tasks where morphological characteristics vary substantially throughout the maturation process.

**Table 5:** Stage-wise detection performance of baseline RT-DETR across five pomegranate maturity stages.

Class	mAP@50	mAP@50-95	Precision	Recall
Average	87.10%	73.34%	91.05%	79.28%
Bud	86.88%	67.60%	92.03%	73.58%
Early-Fruit	89.71%	80.00%	89.68%	84.38%
Flower	91.50%	77.75%	93.90%	80.33%
Mid-Growth	90.76%	76.17%	93.42%	78.99%
Ripe	96.93%	88.09%	96.16%	90.76%

**Table 6:** Stage-wise detection performance of proposed DFMA-DETR across five pomegranate maturity stages.

Class	mAP@50	mAP@50-95	Precision	Recall
Average	90.23%	76.40%	91.63%	82.39%
Bud	88.42%	70.15%	91.34%	78.26%
Early-Fruit	91.18%	81.22%	90.17%	85.74%
Flower	90.89%	78.31%	93.02%	82.15%
Mid-Growth	91.67%	77.94%	92.51%	81.89%
Ripe	95.99%	86.38%	95.11%	83.91%

Comparative analysis between **Tables 5** and **6** reveals that DFMA-DETR achieves notable improvements across most maturity stages, with particularly pronounced enhancements in challenging detection scenarios. The Bud stage exhibits the most significant improvement in Recall metric, increasing from 73.58% to 78.26%, addressing the baseline model’s limitations in detecting small, morphologically indistinct early-stage features. The Mid-Growth stage, which represents a critical transitional phase with complex visual characteristics, demonstrates substantial performance gains with mAP@50–95 improving from 76.17% to 77.94% and Recall increasing by 2.90% from 78.99% to 81.89%. While slight decreases are observed in certain metrics for Flower and Ripe stages, these represent acceptable trade-offs that contribute to overall balanced performance across the complete maturation spectrum, validating the algorithm’s robustness in handling diverse pomegranate growth characteristics and confirming its practical applicability for comprehensive maturity assessment systems.

#### 3.4.2 Different backbone network comparison

To validate the effectiveness of our proposed DFMB-Net backbone network, we designed ablation experiments comparing different backbone networks. Experiments employed identical detection frameworks and training strategies, utilizing RT-DETR-R18 as the baseline model alongside advanced backbone networks including CSwinTransformer, VanillaNet, and RMT for comparative analysis. All models were trained and tested on identical datasets to ensure fairness and comparability of experimental results. Results are presented in **Table 7**.

**Table 7:** Comparison results of different backbone networks.

Model	mAP@50	mAP@P50-95	Precision	Recall	FLOPs	Parameters
Resnet-18 (He et al., 2016)	87.10%	73.34%	91.05%	79.28%	57.0G	19.8M
CSwinTransformer (Dong et al., 2022)	86.80%	73.10%	89.20%	81.80%	91.3G	30.7M
VanillaNet (Chen et al., 2023)	85.40%	71.80%	88.60%	78.20%	166.2G	27.8M
RMT (Fan et al., 2024)	87.50%	74.20%	90.30%	81.90%	61.5G	21.4M
DFMB-Net	87.93%	74.93%	90.80%	80.32%	50.3G	14.7M

Experimental results demonstrate that the proposed DFMB-Net backbone network exhibits excellent performance in both detection accuracy and computational efficiency. Compared to baseline model RT-DETR-R18, DFMB-Net achieved 0.83% improvement in mAP@50 metric and obtained 1.59% significant enhancement in mAP@50–95 metric. When compared with other advanced backbone networks, DFMB-Net not only surpassed CSwinTransformer and VanillaNet in detection accuracy but also demonstrated superior computational efficiency advantages: parameter count was only 14.7M, representing 52.1% and 47.1% reductions compared to CSwinTransformer and VanillaNet respectively; computational complexity was 50.3G FLOPs, representing 69.7% reduction compared to VanillaNet. These results fully validate the effectiveness of DFMB-Net backbone network design, demonstrating its capability to achieve higher detection accuracy while maintaining lower computational costs.

**Figure 10:**
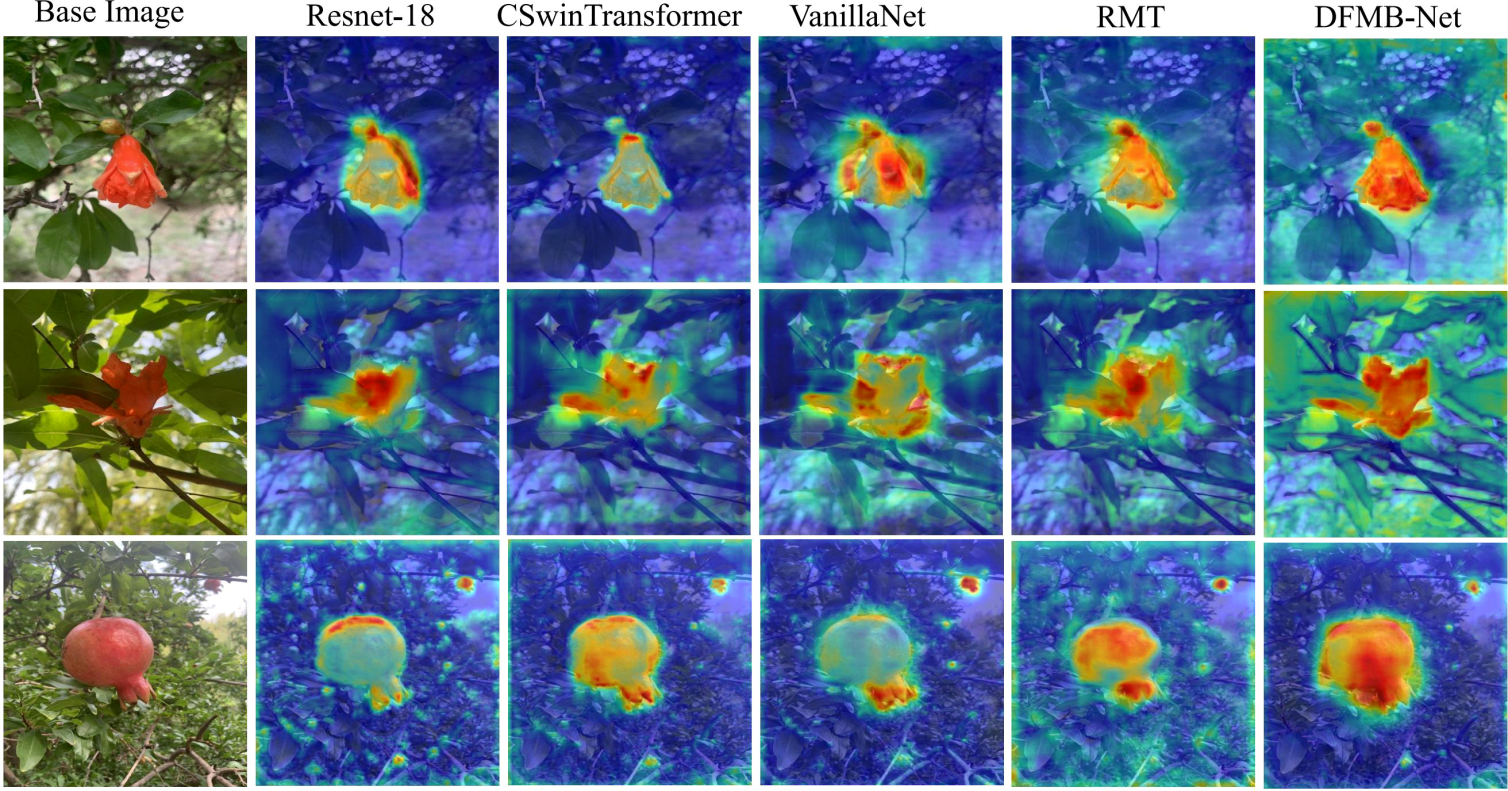
Heat map results of different backbone networks.

To further intuitively demonstrate differences in feature extraction capabilities among various backbone networks, we employed Grad-CAM techniques to generate feature activation heat maps for each backbone network, with results shown in **Figure 10**. Visualization results reveal that the proposed DFMB-Net can more precisely focus on key regions of pomegranate fruits, with concentrated activation areas and high response intensities, particularly in critical areas for maturity discrimination such as pomegranate surface texture variations and color transitions. In contrast, ResNet-18 shows relatively dispersed activation regions, CSwinTransformer and VanillaNet exhibit discontinuous activation when processing local texture details, while RMT network’s feature focusing capability also significantly falls short of DFMB-Net. These visualization results are highly consistent with quantitative experimental data, validating the effectiveness of DFMB-Net dual-domain feature modulation mechanisms in pomegranate maturity feature extraction.

#### 3.4.3 Public dataset comparison experiments

To validate the generalization capability and cross-domain adaptability of our proposed DFMA-DETR algorithm, we conducted public dataset comparison experiments to evaluate algorithm detection performance under different data distributions. Experiments employed the Pomegranate-rjwdq public dataset (Projects Team, 2024) from the Roboflow Universe platform, containing 2,390 high-quality pomegranate images covering various illumination conditions, shooting perspectives, and complex background environments. This dataset exhibits significant differences from our constructed PGSD-5K dataset in image diversity and scene complexity, with experimental results presented in **Table 8**.

**Table 8:** Results of different dataset experiments.

Dataset	Model	mAP@50	mAP@P50-95	Precision	Recall
Pomegranate-rjwdq	RT-DETR	95.73%	68.15%	94.02%	92.76%
	DFMA-DETR	96.86%	70.03%	94.69%	93.84%
PGSD-5K	RT-DETR	87.10%	73.34%	91.05%	79.28%
	DFMA-DETR	90.23%	76.40%	91.63%	82.39%

Experimental results fully demonstrate the excellent performance and superior generalization capability of the DFMA-DETR algorithm on public datasets. Compared to baseline model RT-DETR, DFMA-DETR achieved 1.13% significant improvement in mAP@50 metric and obtained 1.88% performance enhancement in mAP@50–95 metric, while achieving 0.67% and 1.08% improvements in Precision and Recall metrics respectively, validating the feature representation advantages of dual-domain feature modulation backbone networks when processing different data distributions and the precise modeling capabilities of enhanced attention feature fusion modules in complex scenarios. Most importantly, DFMA-DETR demonstrated stable high-precision detection performance on the pomegranate-rjwdq public dataset, not only confirming the technical effectiveness of the proposed innovative architecture but also highlighting the algorithm’s strong robustness when facing cross-domain scene variations and data distribution differences, establishing solid technical foundations for practical engineering applications of pomegranate maturity detection.

#### 3.4.4 Different model comparison experiments

To validate the effectiveness of our proposed DFMA-DETR algorithm, we conducted comparison experiments with mainstream object detection models on the PGSD-5K dataset, with results presented in **Table 9**. Comparison models included traditional two-stage detectors, single-stage detectors, Transformer-based detectors, and RT-DETR series models.

**Table 9:** Comparison results of different models.

Model	mAP@50	mAP@P50-95	Precision	Recall	FLOPs	Parameters
Faster-RCNN (Ren et al., 2016)	85.45%	69.23%	88.21%	81.56%	208.1G	41.4M
Cascade-RCNN (Cai and Vasconcelos, 2018)	86.34%	70.82%	88.97%	82.45%	206.2G	32.3M
YOLOV8m (Sohan et al., 2024)	86.79%	71.56%	89.34%	82.91%	78.7G	28.9M
YOLOV10m (Wang A. et al., 2024)	87.45%	72.34%	90.12%	83.67%	58.9G	15.3M
YOLOV11m (Khanam and Hussain, 2024)	88.23%	73.12%	90.89%	84.34%	67.7G	20.1M
YOLOV12m (Tian et al., 2025)	88.01%	72.89%	90.67%	84.12%	67.2G	20.0M
D-Fine-M (Peng et al., 2024)	87.12%	71.89%	89.56%	83.23%	56.4G	19.2M
DEIM-D-Fine-M (Huang et al., 2025)	87.98%	72.67%	90.45%	83.89%	56.4G	19.2M
RT-DETR-R18 (Zhao et al., 2024)	87.10%	73.34%	91.05%	79.28%	57.0G	19.8M
RT-DETR-R50-tiny (Zhao et al., 2024)	88.56%	74.45%	91.34%	84.78%	134.8G	42.9M
RT-DETR-L (Zhao et al., 2024)	88.73%	74.98%	91.67%	85.12%	108.3G	33.0M
DFMA-DETR	90.23%	76.40%	91.63%	82.39%	51.1G	16.8M

Experimental results indicate that DFMA-DETR achieved significant performance improvements across multiple key metrics. In detection accuracy, DFMA-DETR realized 90.23% mAP@50 and 76.40% mAP@50-95, representing 1.50% and 1.42% improvements over baseline model RT-DETR-L respectively, and 2.00% and 3.28% improvements over the closest-performing YOLOv11m respectively. In computational efficiency, DFMA-DETR achieved optimal accuracy-efficiency balance with only 16.8M parameters and 51.1G computational complexity, reducing parameters by 49.1% compared to RT-DETR-L while maintaining higher detection accuracy.

**Figure 11:**
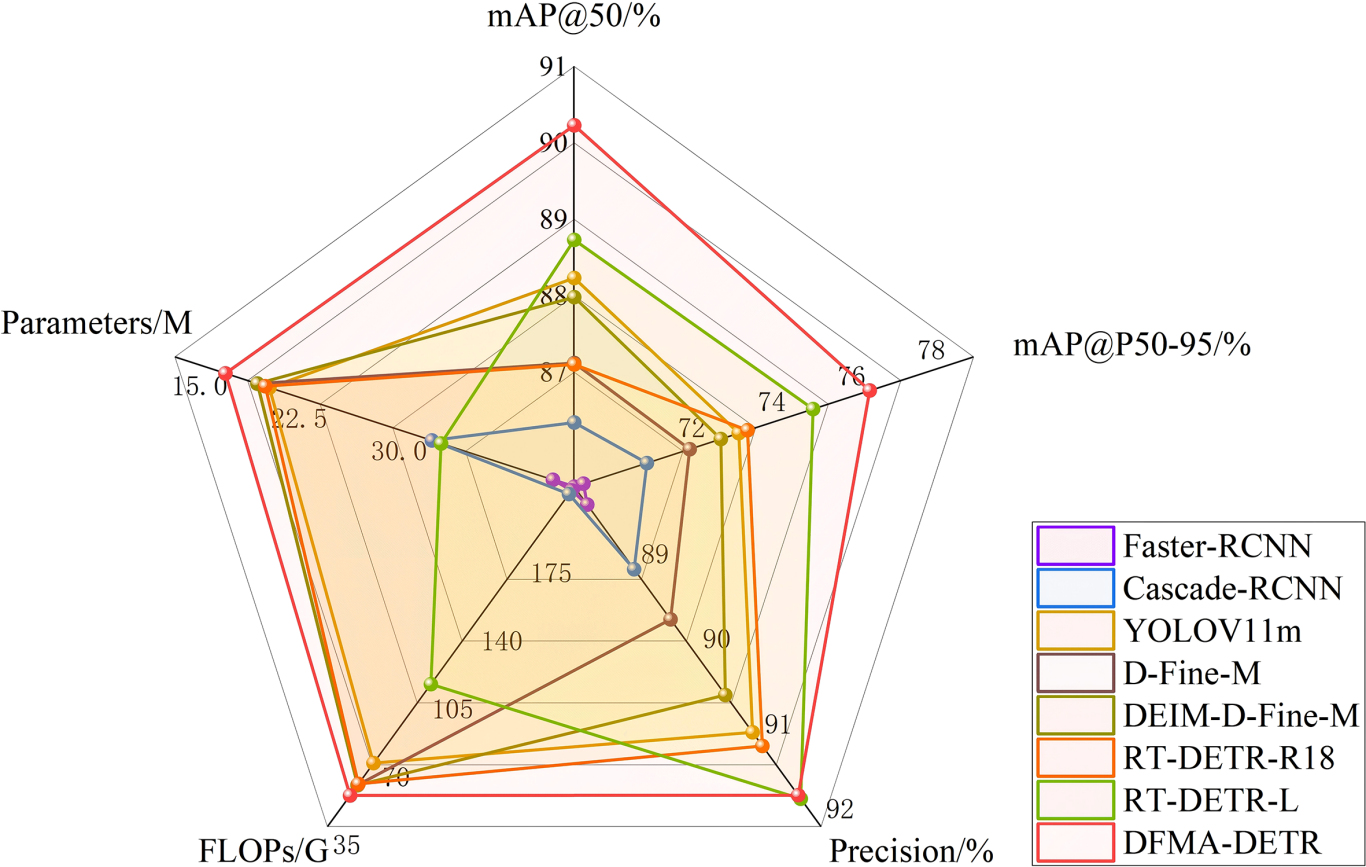
Radar chart of different model comparison experiment results.

Through multi-dimensional performance comparison analysis, the radar chart shown in **Figure 11** clearly demonstrates the comprehensive advantages of DFMA-DETR over mainstream detection models in core metrics including detection accuracy and computational efficiency. Radar chart results reveal that DFMA-DETR maintains high-precision detection capabilities while performing exceptionally well in model lightweighting, with radar chart contours matching optimal models in accuracy metrics while significantly outperforming other comparison models in efficiency metrics, demonstrating the significant advantages of the proposed algorithm in practical applications. Particularly noteworthy is that DFMA-DETR achieved 91.63% in the Precision metric, exhibiting excellent detection accuracy. These results fully validate the effectiveness of our proposed innovative designs including dual-domain feature modulation backbone networks, enhanced attention feature fusion modules, and adaptive interpolation upsampling processors.

### 3.5 Model experimental results visualization

**Figure 12:**
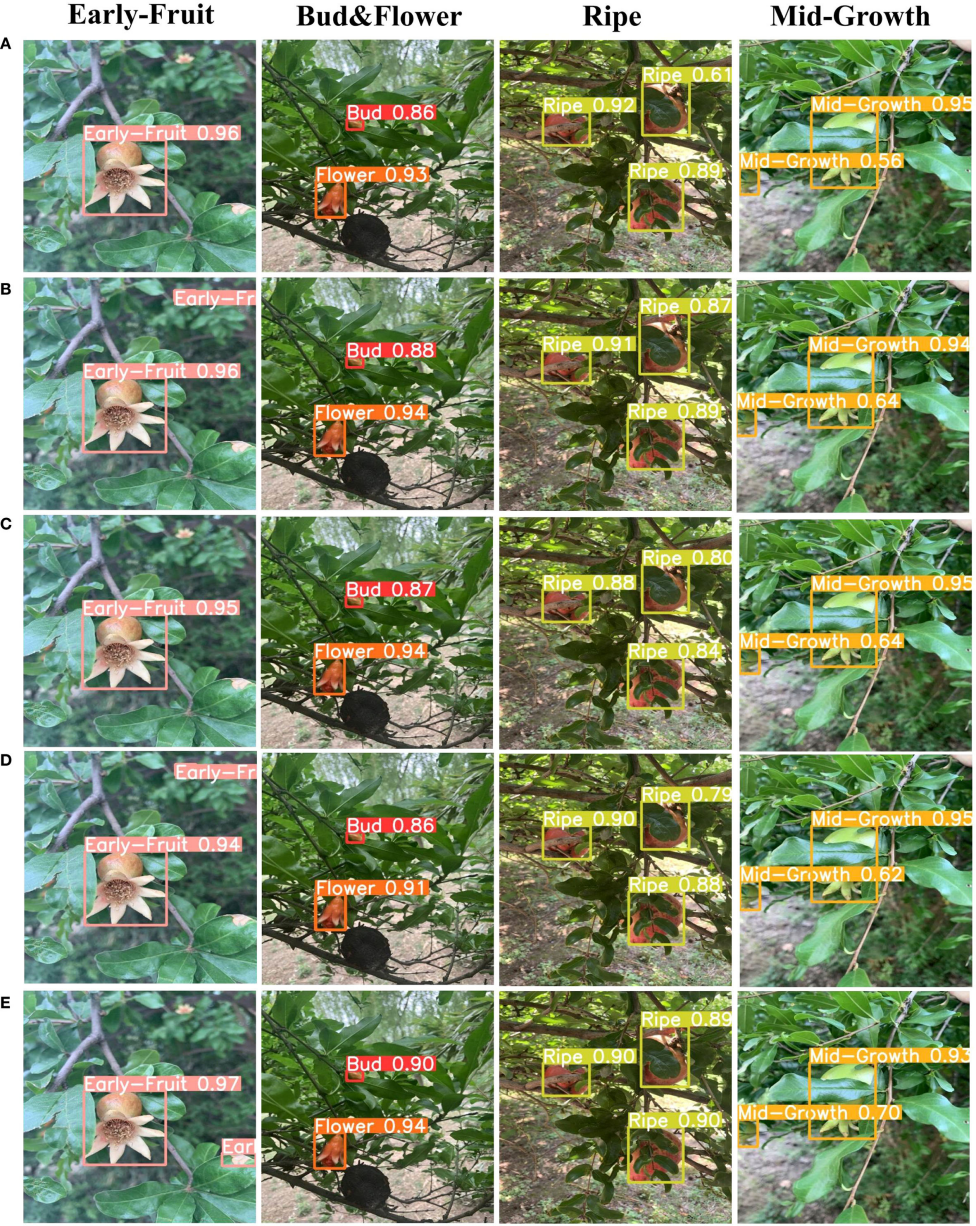
Visualization of different model experimental results, where **(A)** Shows faster-RCNN detection results, **(B)** Shows YOLOV12m, **(C)** Shows DEIM-D-fine-M detection results, **(D)** Shows RT-DETR-R18 detection results, and **(E)** Shows DFMA-DETR detection results.

To intuitively evaluate performance differences among various detection models in pomegranate maturity recognition tasks, we selected representative test samples from the PGSD-5K dataset covering four key maturity stages: Early-Fruit, Bud&Flower, Ripe, and Mid-Growth, presenting comparative detection results of Faster-RCNN, YOLOv12m, DEIM-D-Fine-M, RT-DETR-R18, and our proposed DFMA-DETR algorithm, as illustrated in **Figure 12**. Visualization experiments employed identical input images and detection threshold settings, ensuring fairness among different model comparisons. Each detection result displays predicted bounding boxes, category labels, and corresponding confidence scores, providing intuitive visual evidence for quantitative analysis of detection accuracy and stability across models.

From visualization results, the proposed DFMA-DETR algorithm demonstrates excellent detection performance and higher prediction confidence across all maturity stages. Specifically, DFMA-DETR achieved high confidence of 0.97 in the Early-Fruit stage, significantly surpassing other models; maintained stable confidence levels of 90–94 in the Bud&Flower stage; realized excellent performance of 0.89-0.90 in the Ripe stage; and achieved reliable detection accuracy of 0.70-0.93 in the Mid-Growth stage. In contrast, while traditional algorithms can accomplish basic detection tasks, they clearly fall short of DFMA-DETR in confidence stability. These visualization results fully validate the effectiveness of dual-domain feature modulation backbone networks and enhanced attention feature fusion modules in improving pomegranate maturity detection accuracy and robustness, providing powerful visual evidence for the superiority of the proposed algorithm.

Here’s the English translation of the conclusion section at SCI Q1 level:

## 4 Conclusion

This study addresses critical limitations inherent in conventional pomegranate maturity detection approaches, particularly their inadequate feature representation capabilities, monolithic attention mechanisms, and constrained multi-scale feature fusion performance within complex agricultural environments. We introduce DFMA-DETR, a novel pomegranate maturity detection algorithm that incorporates several key innovations: the DFMB-Net dual-domain feature modulation backbone network, the EAFF enhanced attention feature fusion module, and the AIUP and MFCM optimization components. These elements collectively establish an end-to-end detection framework that integrates spatial-frequency domain collaborative processing, adaptive sparse attention mechanisms, and multi-scale feature adaptation.

Experimental validation demonstrates that DFMA-DETR achieves remarkable performance on the PGSD-5K dataset, attaining 90.23% mAP@50 and 76.40% mAP@50-95, representing improvements of 3.13% and 3.06% respectively over baseline models. Furthermore, the algorithm exhibits excellent generalization capabilities and cross-domain adaptability when evaluated on the publicly available Pomegranate-rjwdq dataset.

Aside from showing improved dramatic detection accuracy, the proposed algorithm is also able to perform an effective trade-off between computational efficiency and detection performance. With only 16.8M parameters and 51.1G FLOPs computational complexity, it provides a solid technical foundation for engineering application in pomegranate maturity assessment. Systematic ablation tests and visual analysis also more effectively promote the effectiveness and cooperative performance of each new module, confirming the advantage of the dual-domain feature modulation mechanism in finding complex surface textures and periodic feature change of pomegranates, and the enhanced attention mechanism’s ability for precise feature localization in cluttered interference background.

This research creates new theoretical foundations and engineering references for intelligent detection technologies in precision agriculture, offering tremendous academic value and prospect application value for pushing forward agricultural modernization and intelligentization.

## 5 Discussion

The DFMA-DETR algorithm, which was recently proposed, shows better performance improvements in pomegranate maturity detection, whose major breakthrough is constructed on the designing of a dual-domain feature modulation mechanism for enabling spatial-frequency collaborative processing with an adaptive sparse attention fusion framework. Compared to traditional single-domain feature extraction-based approaches, our DFMB-Net dual-domain feature modulation backbone network captures pomegranate surface spatial texture details and frequency-domain periodic features simultaneously. This innovation adequately addresses the inherent issue of lacking feature representation capability of traditional convolutional neural networks in coping with complex agricultural scenarios. The dynamic adaptive sparse attention mechanism of the EAFF module achieves optimal trade-off among computational overhead and feature representation capability by dynamic weight adjustment. In contrast to traditional dense attention mechanisms, it lowers computational complexity significantly without sacrificing much accuracy. These findings not only encourage the application of Transformer-based object detection technology in agricultural applications but also create new theoretical models and technical paths for multi-domain feature fusion in severe environments.

Regarding real-time performance capabilities, our DFMA-DETR demonstrates superior inference efficiency in practical deployment scenarios. Performance evaluation on NVIDIA T4 GPU infrastructure reveals that DFMA-DETR achieves approximately 118 FPS during inference, representing a 9.3% improvement over the baseline RT-DETR’s 108 FPS. This enhancement directly results from our optimized architecture design, particularly the reduced computational complexity (51.1G FLOPs compared to 57.0G baseline) and streamlined parameter count (16.8M versus 19.8M parameters).

For edge computing deployment scenarios critical to precision agriculture, DFMA-DETR maintains robust real-time capabilities across constrained computational environments. Testing on NVIDIA Jetson AGX Orin devices demonstrates inference rates of 32–35 FPS, while deployment on resource-limited Jetson Orin Nano platforms achieves stable performance at 16–18 FPS. These metrics enable practical field deployment for autonomous agricultural monitoring systems, where immediate decision-making capabilities are essential for timely intervention in pomegranate cultivation management.

Despite significant achievements, several limitations warrant attention and improvement in future work. First, while the current PGSD-5K dataset encompasses five critical stages of pomegranate growth, samples under extreme illumination conditions, severe occlusions, and dense multi-target scenarios remain relatively limited, potentially affecting model generalization capabilities in more complex and diverse agricultural environments. Second, although the frequency-domain feature processing module FTEU, based on fractional Fourier transform theory, exhibits excellent performance in texture feature extraction, its relatively high computational complexity poses challenges for deployment on resource-constrained edge devices. Additionally, this research primarily focuses on maturity detection for a single pomegranate variety, with insufficient consideration of morphological, color, and textural variations across different cultivars, thereby limiting algorithm universality. Future research should expand dataset scale and diversity, optimize computational efficiency of frequency-domain processing modules, and explore universal pomegranate maturity detection models across varieties and regions.

Looking forward, the dual-domain feature modulation and enhanced attention fusion framework established in this study holds promise for further expansion and deepening across multiple directions. First, this framework could be extended to maturity detection tasks for other fruit crops, utilizing transfer learning and domain adaptation techniques to develop universal detection models across crop species, providing broader technical support for precision agriculture intelligentization. Second, integration with multimodal sensing technologies, including near-infrared spectroscopy, hyperspectral imaging, and three-dimensional point cloud data, could establish comprehensive detection systems based on multi-source information fusion, potentially enabling holistic assessment of both internal and external fruit quality. Furthermore, with rapid developments in edge computing and model compression technologies, future research could explore lightweight dual-domain feature modulation network designs, developing real-time detection systems suitable for mobile platforms such as unmanned aerial vehicles and robots. Finally, incorporating reinforcement learning and active learning strategies could construct adaptive online learning frameworks, enabling detection models to continuously optimize and improve during practical applications, providing essential technical foundations for achieving truly intelligent agricultural production.

## Data Availability

The raw data supporting the conclusions of this article will be made available by the authors, without undue reservation.
